# Microstomia is associated with functional impairment and is a poor prognostic factor in systemic sclerosis – a single center observational study with survival analysis

**DOI:** 10.1186/s12903-024-05178-6

**Published:** 2024-11-15

**Authors:** Kristóf Filipánits, Gabriella Nagy, Cecília Varjú, László Czirják, Tünde Minier

**Affiliations:** Department of Rheumatology and Immunology, Medical School, University of Pécs, 1 Akác street, Pécs, H-7632 Hungary

**Keywords:** Systemic sclerosis, Mouth opening ability, Microstomia, Disability, Survival

## Abstract

**Background:**

Objectives were to assess the mouth opening ability (MOA) among patients with systemic sclerosis (SSc) in comparison to a healthy control population. The impact of microstomia (decrease in any of the MOA parameters) on physical performance and long-term survival was also investigated.

**Methods:**

Interincisal (ID), interlabial (LD), the intercommissural distances at both opened mouth and closed mouth (OW, CW) and the oral area (OA) and circumference (OC) all were assessed in 131 SSc patients. Microstomia was defined based on the oral aperture values of a healthy control group (*n* = 63) with similar median age and sex distribution. Tests evaluating functional performance were performed. Survival analysis by univariate and multivariate Cox regression analysis was performed.

**Results:**

Microstomia was present in 56.5% of the entire SSc cohort. Patients with microstomia had higher number of contractures compared to those with preserved oral aperture parameters (median 10 vs. 7, *p* < 0.01). LD, OA and OC correlated negatively with the number of joint contractures, both in the entire SSc cohort and separately in the diffuse (dcSSc) and limited cutaneous subsets (lcSSc), (rho: between − 0.267 and − 0.516, *p* < 0.05). Reduced ID was associated with worse Health Assessment Questionnaire Disability Index (HAQ-DI) only in dcSSc while reduced LD was linked to increased HAQ-DI scores in both SSc subsets. Decreased OA upon enrollment was associated with an increased risk in mortality by multivariate Cox regression analysis (HR: 2.74; 95% CI, 1.15–6.53).

**Conclusions:**

Microstomia was associated with joint damage, and higher overall disability based on HAQ-DI. Interlabial distance was a beneficial, convenient measurable parameter to characterize oral aperture. Oral area was an independent poor prognostic factor regarding long-term survival.

**Supplementary Information:**

The online version contains supplementary material available at 10.1186/s12903-024-05178-6.

## Background

Systemic sclerosis (SSc) is a multisystem disorder characterized by autoimmune phenomena, vasculopathy, and tissue fibrosis [1]. SSc is associated with the highest mortality rates among connective tissue diseases due to the various internal organ manifestations [2, 3]. Additionally, the disease is associated with impairment of functional ability, pain, fatigue and change in facial features which can strikingly impact the quality of life [4, 5]. Internal organ involvements along with skin and musculoskeletal features of the disease also result in significantly decreased quality of life (QoL) [6]. Various poor prognostic factors for disease worsening and mortality were identified in SSc studies: older age upon disease onset, male gender, pulmonary, cardiac and renal involvement, and a higher baseline Health Assessment Questionnaire Disability Index (HAQ-DI) score, coexistent malignancy and musculoskeletal involvement, as in the case of small joint contractures, and a history of arterial hypertension [5, 7–12].

In clinical practice, SSc is divided into two subtypes, diffuse cutaneous systemic sclerosis (dcSSc) and limited cutaneous systemic sclerosis (lcSSc), the latter being associated with better life expectancy [13, 14].

Decreased mouth opening ability (MOA), or microstomia, is present in approximately 7–80% of SSc patients [15–17], the large variations being due to the various investigated parameters and definitions. The measurement of interincisal distance (ID) was most frequently used to compare the MOA between SSc patients and healthy controls (HCs) [17–21]. Various arbitrary values were used as cut-off values of decreased ID in different studies (e.g., ID < 30 mm, 40–43 mm) ([17, 22–26]. Vertical interlabial distance at maximally opened mouth (LD), and intercommissural width at open as well as closed mouth (OW and CW) were also previously studied [16, 27, 28].

Since there is no consensus regarding the objective assessment of the oral aperture parameters in SSc, ID, LD, OW and also CW all were chosen as outcome measures towards evaluating the efficacy in different pharmacological treatment interventions or physiotherapy and occupational therapy [26–31].

Regarding the natural course and clinical relevance of microstomia, a recently published large Dutch cohort tallying 382 SSc patients showed microstomia, defined as ID < 30 mm, was associated with more extended skin involvement, vasculopathy, gastrointestinal, pulmonary and renal involvement [17]. Although microstomia was infrequent among this patient cohort (7%), mouth opening was stable in the majority of SSc patients during the two-year follow-up period, an annual decrease of > 5 mm in MOA was accompanied by increase in disease severity [17]. Another study referencing the French national SSc cohort demonstrated decreased ID defined also based on ID measurement can be used to predict disease severity and survival in dcSSc [24]. Decreased MOA has also been found to have a remarkable impact upon the QoL in SSc [32].

The main objectives of this study were to systematically assess the currently used tools regarding the evaluation of MOA in SSc and to assess the significance of MOA parameters in the long-term follow-up.

## Methods

### Study design and clinical data

Prospective study with one hundred and thirty-one consecutive Caucasian SSc patients from a single tertiary care center was carried out. Diagnosis of SSc and classification into diffuse or limited subset was performed based on the criteria proposed by LeRoy and Medsger [13].

According to a retrospective analysis, these patients also fulfilled the 2013 ACR/EULAR SSc classification criteria [33]. Demographical, clinical and laboratory parameters were collected according to our standard protocol [34, 35].

Disease duration in years was estimated based on the elapsed time between the onset of the first non-Raynaud symptom of SSc and the date of the baseline investigation.

Clinical characteristics of the patient population were described in detail in a previously published paper of the research group [36].

Disease activity was assessed using the preliminary European Scleroderma Study Group activity index, available upon study entry (EScSG-AI), and the scleroderma activity index derived from the EScSG-AI (Pecs-AI) [36, 37]. The Pecs-AI attempted to enhance the objective information related to lung, vascular and skin involvement. A musculoskeletal examination was also performed based on a pre-defined local protocol [38]. Peripheral joint contracture (CC) was diagnosed if there was a 25% decrease in range of motion (ROM) in at least one joint-movement direction. Extensive contracture (extCC) was defined as a decrease of greater than 50% in ROM.

The global functional impairment was assessed using the Health Assessment Questionnaire (HAQ-DI) [39]. The hand anatomic index (HAI) was used to quantify hand deformities [40].

In consideration of HCs, sixty-three healthy Caucasian volunteers with no systemic autoimmune disease in their case history and similar median age and sex distribution were admitted to determine the lower limit of normal (LLN) MOA.

### Oral aperture parameters

**Fig. 1:**
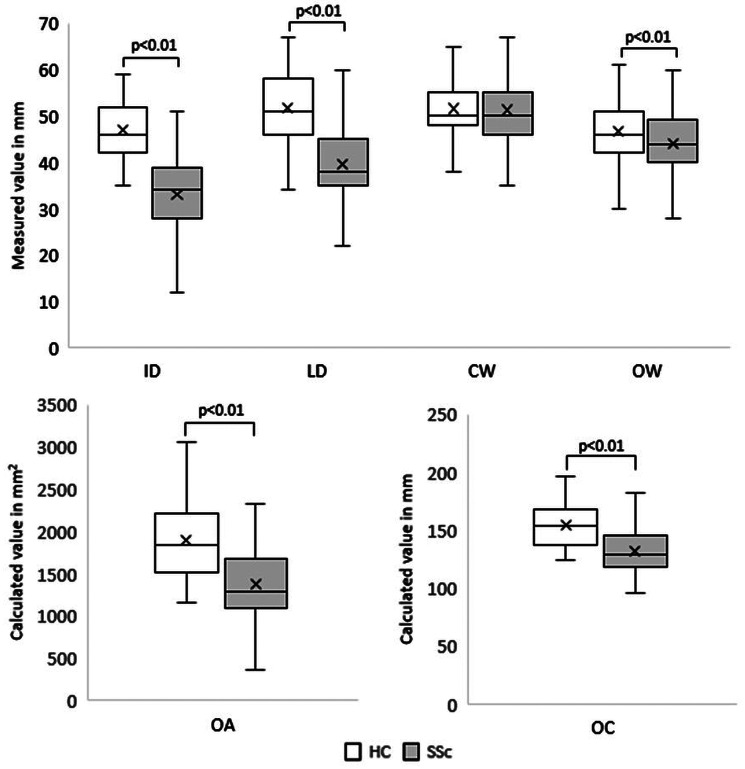
Comparison of oral aperture parameters of systemic sclerosis patients (*n* = 131) and the healthy control group (*n* = 63) upon enrolment. Legend: ID: vertical interincisal distance at maximally opened mouth; LD: vertical interlabial distance at maximally opened mouth; OW: horizontal width at maximally opened mouth; CW: horizontal width at closed mouth; OA: oral area; OC: oral circumference; SSc: systemic sclerosis patients; HC: healthy controls; mm: millimetres. Statistical test used: Mann-Whitney U test; level of significance: *p* < 0.01

ID, LD , OW and CW were measured in millimeters by two experienced physical therapists with a disposable ruler. When considering the mouth by good approximation resembles the shape of an ellipse, two parameters, the oral circumference (OC) and oral area (OA), were also generated [see Fig. 1 in Additional File 1].

Based on the ID, LD, OW, CW, OA and OC values of HCs, we defined the LLN as mean–2SD values. Values below the LLN among SSc patients were considered as decreased values and consequently referred to as microstomia.

We identified the following LLN values of MOA: 33 mm for ID, 37 mm for LD, 38 mm for CW, 34 mm for OW, 941 mm^2^ for OA and 115 mm for OC.

### Survival analysis

Survival data were calculated based on the available data of patients on 15 March, 2020, to avoid the negative impact of the Coronavirus 2019 (COVID-19) pandemic [41]. Causes of death were determined based on final reports, consultation with the patients’ GP and autopsy results.

### Statistical analysis

Central tendency and dispersion for continuous data were described using mean and standard deviation (± SD) or median and interquartile range (IQR). Categorical variables were presented with frequencies and percentages. Non-parametric tests were used for comparison between subgroups (Mann-Whitney test and the Spearman’s correlation for continuous data) based on the distribution of different variables. To effectively investigate factors affecting survival, Kaplan-Meier and Cox Proportional Hazards Model analysis with 95% confidence intervals (CI) were used. SPSS 28.0 for Windows (SPSS Inc., Chicago, IL, USA) was used for all analyses.

Each participant freely submitted their written informed consent according to the Declaration of Helsinki. Ethical approval was provided by the Regional and Institutional Research Ethics Committee, Medical Center, University of Pécs (Approval No. 2720/2006 and 4906/2013) and the Hungarian National Ethics Committee (IF-6720-6/2015.).

## Results

### Study population

One hundred and thirty-one consecutive Caucasian patients (119 females and 12 males) were enrolled. Forty-one patients had belonged to the dcSSc and 90 patients to the lcSSc subtype. Their median age was 57 years (IQR: 50;64) with median disease duration consisting of 6 years (IQR: 3;11) at the time of enrollment.

Sixty-three HCs were included in the analysis. The median age upon enrollment was 50 (38;68) years, in which 8 out of 63 volunteers (12.6%) were male. There was no significant difference between the age of SSc patients and healthy controls based on the Mann-Whitney U test (*p* > 0.05).

#### Oral aperture parameters of healthy controls and SSc patients

##### Prevalence of microstomia in SSc patients

Except CW, HCs had significantly higher MOA values when compared with SSc patients upon enrollment (Fig. 1).

Microstomia defined as values lower than the mean, -2SD of HCs in any of the six investigated oral aperture parameters was present in 56.5% of the entire SSc cohort. 46% (*n* = 59) of the SSc patients had decreased ID, 37% (*n* = 49) had decreased LD, 2% (*n* = 3) had decreased CW, 5% (*n* = 6) had decreased OW, 10% (*n* = 13) had decreased OA and 19% (*n* = 25) had decreased OC. Patients with decreased OA had significantly lower ID values when compared to patients with preserved OA yet decreased ID values /median (IQR) ID: 20 (14;25) mm vs. 29 (25;30) mm, *p* < 0.01/.

##### Sex

Each parameter of the mouth opening among females proved significantly lower when compared to males in the HCs [see Table 1 in Additional file 1].

Female SSc patients had only lower CW, OA, and OC values when compared to males. Female SSc patients and male SSc patients separately had lower MOA values except for CW, when compared to HCs with same sex.

##### SSc subtype

The only significant difference in oral aperture values when comparing dcSSc and lcSSc patients was found in the ID, which was lower among dcSSc patients/median (IQR) 30 (22;37) vs. 35 (30;40), *p* < 0.01/. Although a decreased ID was more prevalent among dcSSc patients, decreased LD and OA based on the cut-off values previously defined (see Materials and Methods) were similarly frequent in both the diffuse and limited SSc.

##### Disease duration

Disease duration in the entire SSc group and in dcSSc showed no correlation with any of the MOA parameters. Only the ID showed a weak correlation with disease duration among the lcSSc group (rho: − 0.235, *p* < 0.05). Grouping SSc patients by disease duration (≤ 2 years vs. >2 years), ID, LD and OC were lower in the entire SSc, dcSSc, and lcSSc groups with early disease compared to HCs. There were no differences in ID, LD, OW and CW values between SSc patients with early disease and those with longer disease duration. The OA and OC were lower among SSc patients with > 2 years disease duration.

Although microstomia was more frequent among SSc patients with disease duration > 2 years, a significant percentage of early SSc patients had also decreased MOA (61% vs. 37%, *p* < 0.05). Decreased ID was already present among early SSc patients (*n* = 10/29, 34.5%), being more common in dcSSc (6/10 vs. 3/19, *p* < 0.05). Decreased LD occurred in 24.1% (*n* = 7/29) among early SSc patients.

#### Oral aperture and clinical parameters

Among SSc patients with disease duration > 2 years (*n* = 102), ESR showed a weak negative correlation with ID (rho: − 0.203, *p* < 0.01). The ID among dcSSc patients with disease duration > 2 years (*n* = 32) showed a moderate correlation with BMI (rho: 0.445, *p* = 0.011) and moderate negative correlation with mRSS (rho: − 0.423, *p* = 0.016). Moderate negative correlations were observed between LD and ESR (rho: − 0.492, *p* = 0.04), also between OA and ESR (rho: 0.430, *p* = 0.014) among dcSSc patients with longer disease duration. No other significant correlations of MOA parameters and internal organ involvement were noted in the entire SSc cohort, the dcSSc and lcSSc subsets.

Patients with microstomia had higher number of contractures compared to those with preserved oral aperture parameters (median 10 vs. 7, *p* < 0.01, Mann-Whitney U test).

When analysing the MOA parameters separately, SSc patients with decreased ID, LD or OC had more contractures than when contrasted to patients with preserved MOA values. The number of extensive CCs was higher among patients if any of the ID, LD, OA or OC parameters were decreased (Table 1).

**Table 1:** Measures of structural joint involvement and global disability in patients with systemic sclerosis with decreased and preserved oral aperture parameters upon enrollment (*n* = 131)

All SSc patients (*n* = 131)									
	ID		LD		OA			OC	
Median (IQR)	Preserved (*n* = 68)	Decreased (*n* = 59)	Preserved (*n* = 82)	Decreased (*n* = 49)	Preserved (*n* = 117)	Decreased (*n* = 13)		Preserved (*n* = 105)	Decreased (*n* = 25)
CC number	**7 (6;9)**	10 (6;13)**	**7 (6;10)**	11 (7;15)***	8 (6;12)	13 (5;19)		**8 (6;11)**	12 (6;15)*
extCC number	**0 (0;3)**	3 (1;7)***	**0 (0;3)**	4 (1;8)***	**1 (0;4)**	4 (0;11)*		**1 (0;4)**	3 (0;10)*
HAI	2.4 (1,7;3.2)	1.9 (1.5;3)	**2.6** **(1.8;3.3)**	**1.7** (1.2;2.3)***	**2.2** **(1.6;3.1)**	**1.42** (0.6;2.7)*		**2.27** **(1.7;3.2)**	1.68 (1.1;2.4)*
HAQ-DI	**0.88 (0.16;1.47)**	1.13 (0.63;1.88)**	**0.75** **(0.13;1.38)**	**1.5** **(1.06;2.0)** ***	**1** **(0.38;1.50)**	1.75 (1.44;2.19)***		**0.88** **(0.25;1.5)**	1.5 (1.19;2.0)***
SSc patients with disease duration > 2 years (*n* = 102)									
	ID		LD		OA			OC	
Median (IQR)	Preserved (*n* = 49)	Decreased (*n* = 49)	Preserved (*n* = 60)	Decreased (*n* = 42)	Preserved (*n* = 90)		Decreased (*n* = 12)	Preserved (*n* = 79)	Decreased (*n* = 25)
CC number	**7 (6;9)**	10 (6;13)*	**7 (6;9)**	12 (7;15)***	**8 (6;12)**		14 (6;19)*	**8 (6;12)**	12 (7;15)*
extCC number	**0 (0;3)**	3 (1;7)**	**0 (0;2)**	4 (1;8)***	**1 (0;4)**		7 (1;11)*	**1 (0;4)**	4 (0;10)*
HAI	2.4 (1.7;3.3)	1.8 (1.4;2.9)	**2.6** **(1.8;3.4)**	**1.6** **(1.2;2.0)** ***	**2.1** **(1.6;3.2)**		**1.4** (0.6;2.8)*	**2.2** **(1.6;3.3)**	1.7 (0.8;2.5)*
HAQ-DI	**0.88 (0.38;1.5)**	1.13 (0.75;1.88)*	**0.88 (0.25;1.38)**	**1.5** (1.13;2.0)***	**1.0** **(0.5;1.5)**		**1.75** (1.4;2.28)**	**1.0** **(0.38;1.5)**	1.5 (1.13;2.0)**

Legend ID: vertical interincisal distance at maximally opened mouth; LD: vertical interlabial distance at maximally opened mouth, CW: horizontal width at closed mouth; OW: horizontal width at maximally opened mouth; OA: oral area; OC: oral circumference; CC: contracture, defined by limitation in range of motion in any joint greater than 25% of the normal range evaluated by physiotherapists; extCC: extensive contracture, defined by limitation in range of motion in any joint greater than 50% of the normal range evaluated by physiotherapists. HAI: Hand Anatomic Index, HAQ-DI: Health Assessment Questionnaire Disability Index. Based on the ID, LD, OW, CW, OA and OC values of healthy controls, we defined the lower limit of normal values (LLN) as mean–2SD values. Decreased values, i.e. below the LLN, are below 33 mm for ID, 37 mm for LD, 38 mm for CW, 34 mm for OW, 941 mm2 for OA, and 115 mm for OC. Bold characters represent significant differences between the investigated parameters at baseline (Mann-Whitney U test, *p < 0.05, **p < 0.01, ***p < 0.001)

Correlation analysis between vertical and calculated oral parameters (ID, LD, OA and/or OC) and the number of CCs, as well as extCCs, confirmed these relationships (rho between − 0.279 and − 0.394, *p* < 0.01) (Table 2).

**Table 2:** Correlations between oral aperture parameters and measures of structural joint involvement and global disability in 131 systemic sclerosis patients upon enrollment

All SSc patients (*n* = 131)												
		ID			LD			OA			OC	
CC		− 0.279**			− 0.328***			− 0.345***			− 0.315***	
extCC		− 0.394***			− 0.392***			− 0.393***			− 0.372***	
HAI		0.234**			0.374***			0.405***			0.370***	
HAQ-DI		− 0.316***			− 0.457***			− 0.475***			− 0.461***	
Grouping by disease duration												
	≤ 2 Years (SSc *n* = 29, dcSSc *n* = 9, lcSSc *n* = 20)						> 2 Years (SSc *n* = 102, dcSSc *n* = 32, lcSSc *n* = 70)					
	ID		LD	OA		OC	ID		LD	OA		OC
CC	− 0.236		− 0.056	− 0.069		− 0.067	− 0.288**		− 0.404**	− 0.393***		− 0.346***
extCC	− 0.278		− 0.102	− 0.211		− 0.206	− 0.431***		− 0.470***	− 0.448***		− 0.412***
HAI	− 0.006		0.100	0.294		0.284	0.296**		0.437***	0.433***		0.382***
HAQ-DI	− 0.463*		− 0.386*	− 0.502**		− 0.508**	− 0.269**		− 0.449**	− 0.441***		− 0.420***

Legend ID: vertical interincisal distance at maximally opened mouth; LD: vertical interlabial distance at maximally opened mouth; OA: oral area; OC: oral circumference; CC: contracture, defined by limitation in range of motion in any joint greater than 25% of the normal range evaluated by physiotherapists; extCC: extensive contracture, defined by limitation in range of motion in any joint greater than 50% of the normal range evaluated by physiotherapists; HAI: Hand Anatomic Index; HAQ-DI: Health Assessment Questionnaire disability index. Bold characters represent significant correlations between the investigated parameters at baseline (Spearman correlation, *p < 0.05, **p < 0.01, ***p < 0.001)

Among dcSSc patients, decreased parameters of ID, LD, OA and OC were noted, whereas among lcSSc patients, only decreased LD was associated with a higher number of CCs. Patients afflicted with dcSSc had more frequent extCCs if their ID or OA values were decreased. Reduced LD was also associated with a higher number of extCCs among lcSSc patients. Correlation analysis also confirmed these relationships separately in dcSSc and lcSSc subgroups.

Patients with short disease duration showed no association between the number of CCs, number of extCCs and any of the MOA parameters. Patients with > 2 years disease duration had more CCs and extCCs, if any of the investigated MOA parameters were decreased (Table 1). Decreased LD was associated with more CCs and extCCs in both SSc subsets in the case of disease duration > 2 years (data not shown). SSc patients with > 2 years duration (*n* = 102) showed weak to moderate negative correlations between ID, LD, OA and/or OC values and CC as well as extCC number (rho between − 0.288 and − 0.470, *p* < 0.05). LcSSc patients (*n* = 70) with disease duration > 2 years also showed weak to moderate negative correlations between LD, OA and/or OC values and the number of CCs and extCCs (rho between-0.267 and − 0.471, *p* < 0.05) (Table 2).

Hand anatomic index showed worse hand function in patients with microstomia compared to patients with preserved oral aperture (median/IQR/: 1.81/1.39;2.9/ vs. 2.55/1.81;3.23/, *p* < 0.05).

When analysing MOA parameters separately, decreased LD, OA and/or OC was associated with lower HAI, meaning more pronounced impaired range of motion in small hand joints upon enrollment. DcSSc patients with decreased OA had lower HAI /median (IQR) 0.6 (0.3;2.0) vs. 1.8 (1.4; 2.5), *p* < 0.05)/, while among lcSSc patients decreased LD was associated with lower HAI values at baseline /median (IQR) 1.7 (1.1;2.6) vs. 2.8 (2.0;3.36) *p* < 0.05)/. Patients with short disease duration showed no association between decreased MOA and HAI, however, those with > 2 years disease duration and decreased LD, OA and/or OC had lower HAI (Table 1). Correlation analysis confirmed this relationship (Table 2).

#### Oral aperture and measures of functional performance

Patients with decreased ID, LD, OA or OC had higher HAQ-DI scores upon enrollment, reflecting higher overall disability. Reduced ID was only associated with worse HAQ-DI among dcSSc, reduced LD and OC with worse overall disability in both the lcSSc and dcSSc subgroups, whereas OA was associated with worse disability in the lcSSc subset, yet also showed borderline significance among dcSSc (*p* = 0.051). Patients with > 2 years disease duration upon enrollment had higher HAQ-DI, if any of the previous four MOA measures were decreased (Table 1). Patients with early disease and moderate to severe disability (HAQ-DI > 1; *n* = 13/29) had lower OA and OC values when compared to SSc patients with HAQ-DI ≤ 1 /OA median (IQR): 1287 (1106;1544) vs. 1593 (1384;1962), *p* < 0.05 and OC median (IQR): 127 (120;139) vs. 148 (131;157), *p* < 0.05)/.

HAQ-DI showed weak to moderate negative correlation with ID, LD, OA and OC (rho between − 0.316 and − 0.475, *p* < 0.01) in the entire SSc patient cohort. Correlation was moderate to strong negative between MOA parameters and HAQ-DI among the dcSSc subset upon enrollment (rho between − 0.477 and − 0.679, *p* < 0.05). Only LD, OA and OC correlated weakly negative with HAQ-DI in the case among lcSSc patients (rho between − 0.371 and -. 395, *p* < 0.01). HAQ-DI showed weak to moderate negative correlation with ID, LD, OA, and OC among patients with disease duration ≤ 2 years upon enrollment (SSc *n* = 29, dcSSc *n* = 9, lcSSc *n* = 20; rho between − 0.386 and − 0.508, *p* < 0.05). Among dcSSc patients with > 2 years disease duration, OA and OC showed strong negative correlations with HAQ-DI (rho: − 0.653 and − 0.697, *p* < 0.05) (Table 2).

#### Oral aperture and disease activity

Based on the EScSG-AI upon enrollment, 28 patients/21%, median (IQR) EScSG-AI: 2.0 (0.5;3.0)/ were afflicted with an active disease (EScSG-AI ≥ 3.0). According to Pecs-AI, 60 patients/46%, median (IQR) Pecs-AI 2.0 (1.0;3.5)/ showed disease activity (Pecs-AI ≥ 2.5) upon enrollment. Active SSc based on Pecs-AI was more common among patients with decreased LD at baseline (57% vs. 39%, *p* < 0.05). Decreased LD occurred more often among patients with > 2 years disease duration and an active disease (57% vs. 32%, *p* < 0.05). In consideration of subset analysis, this finding was detectable exclusively among lcSSc patients with a disease duration > 2 years (58% vs. 33%, *p* < 0.05). The Pecs-AI has shown weak significant negative correlation with ID, LD, OA and OC (rho between − 0.181 and − 0.273, *p* < 0.05) in the entire patient cohort, whereas no such correlation was observed regarding the EScSG-AI. Upon subset analysis, only lcSSc patients showed weak negative correlation between Pecs-AI, ID and LD (rho: − 0.232 and − 0.292, *p* < 0.05), primarily among those with > 2 years duration upon subset analysis (rho between − 0.332 and − 0.360, *p* < 0.01).

#### Survival analysis

Fifty-one patients succumbed during the follow-up period and 23 patients had been lost to follow-up. The 10-year survival was 78% among dcSSc and 94% in lcSSc, however, there was a relatively rapid decrease in survival afterwards in the dcSSc subset with an approximately 20% decline within 5 years (79.4%, 58.5% and 85.6% at 15 years in the entire SSc, dcSSc and lcSSc groups, respectively, and 74.8%, 56.1% and 80% at 20 years in the entire SSc, dcSSc and lcSSc groups, respectively).

**Fig. 2:**
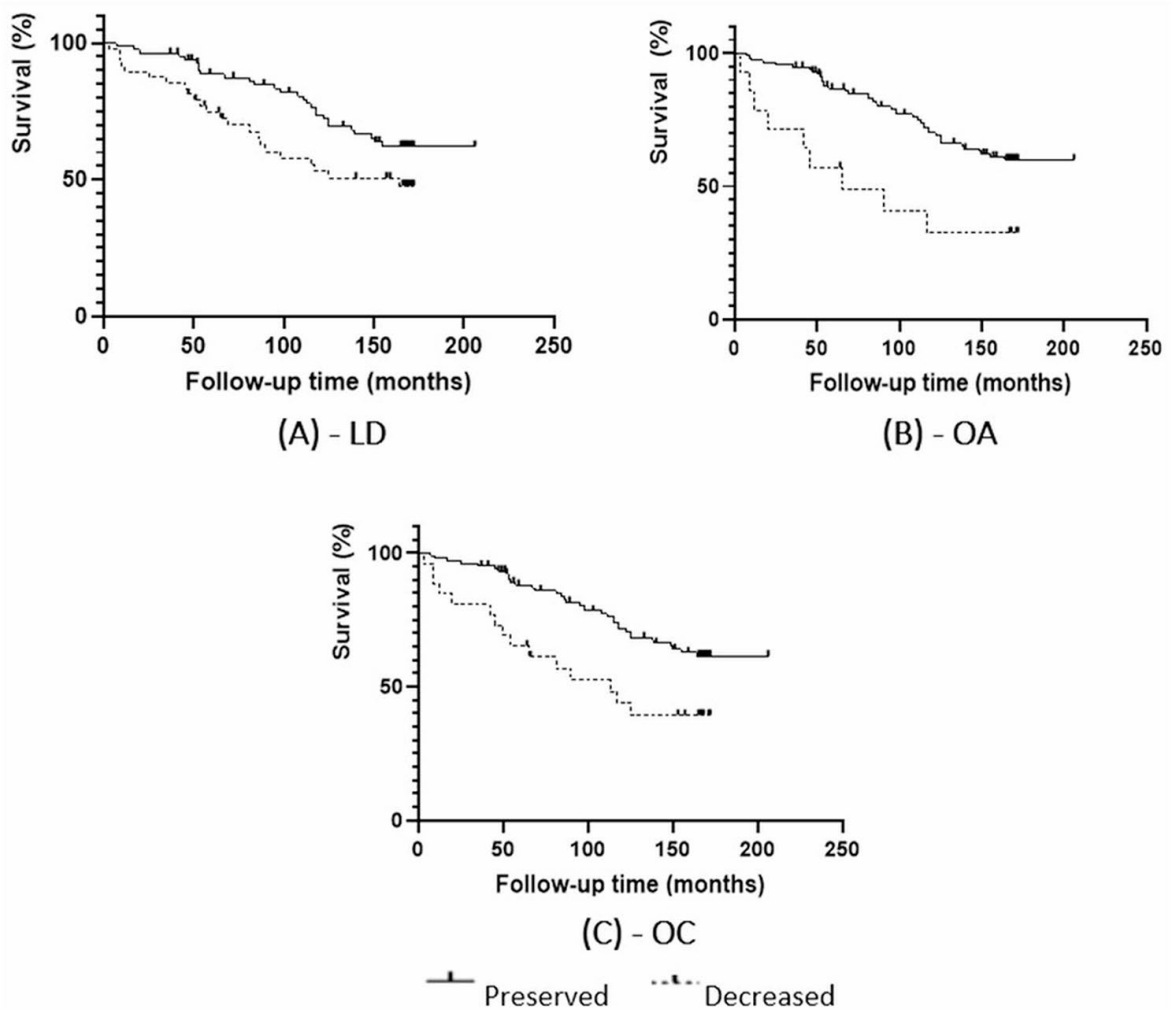
Kaplan-Meier survival analysis of patients with systemic sclerosis based on interlabial distance (**A**), oral area (**B**), and oral circumference (**C**) measurements upon enrollment (*n* = 131 patients). Legend: LD: vertical interlabial distance at maximally opened mouth (decreased: LD < 37 mm); OA: oral area (decreased: OA < 941mm2); OC: oral circumference (decreased: OC < 115 mm). Kaplan-Meier survival analysis, level of significance: p < 0.05. Log Rank chi square (**A**): 4.046, p < 0.05; Log Rank chi square (**B**): 8.787, p < 0.01; Log Rank chi square (**C**): 7.641, p < 0.01

Based on the Kaplan-Meier survival analysis, the following poor prognostic factors were identified: dcSSc subgroup, male sex, forced vital capacity (FVC) < 80%, Diffusing Capacity Of The Lungs For Carbon Monoxide (DLCO) < 80%, HAQ-DI score ≥ 1, low haematocrit (female < 35%, male < 40%), ESR > 30 mm/h, coexistent malignancies and malignancies diagnosed during follow-up, decreased LD, decreased OA and decreased OC upon enrollment (Table 3). Figure 2 shows Kaplan-Meier curves of MOA parameters. A non-significant trend in reference to BMI < 18.5 and low albumin was also observed (Table 3).

**Table 3:** Univariate and multivariate survival analysis of 131 systemic sclerosis patients

Risk factor	Overall mortality risk	*p*
**Univariate survival analysis (Kaplan-Meier)**		
	Log Rank chi-square	
ESR > 30 mm/h	18.853	< 0.001
Low haematocrit (female < 35%, male < 40%)	11.888	< 0.001
Malignancies	11.156	< 0.01
Decreased OA	8.787	< 0.01
Decreased OC	7.641	< 0.01
HAQ-DI score ≥ 1	6.926	< 0.01
FVC < 80%	6.855	< 0.01
Male sex	6.617	< 0.05
DLCO < 80%	6.148	< 0.05
DcSSc subgroup	5.428	< 0.05
Decreased LD	4.046	< 0.05
BMI < 18.5	3.790	0.05
Low albumin	3.742	0.05
**Multivariate survival analysis (Cox Proportional Hazards Model)**		
	HR (95% CI)	p
Male sex	4.171 (1.668–10.430)	< 0.01
Decreased oral area (< 941 mm^2^)	2.742 (1.152–6.530)	< 0.05
ESR > 30 mm/h	2.604 (1.325–5.118)	< 0.01
HAQ-DI	2.019 (1.313–3.105)	< 0.01
Age at disease onset	1.039 (1.015–1.063)	< 0.01
DLCO%	0.978 (0.958–0.997)	< 0.05
BMI	0.913 (0.852–0.978)	< 0.05

Legend ESR: erythrocyte sedimentation rate; malignancies: coexistent malignancies and malignancies diagnosed during follow-up; OA: oral area (decreased: OA < 941mm2); OC: oral circumference (decreased: OC < 115 mm); LD: vertical interlabial distance at maximally opened mouth (decreased: LD < 37 mm); HAQ-DI: Health Assessment Questionnaire - Disability Index; FVC: forced vital capacity; DLCO: diffusing capacity of the lungs for carbon monoxide; BMI: Body Mass Index in kg/m^2^

Based on the multivariate logistic regression analysis, decreased OA upon enrollment was associated with higher risk for death among SSc patients related to all-cause mortality (HR: 2.74; 95% CI, 1.15–6.53) during the follow-up. Male sex, ESR > 30 mm/h, and higher HAQ-DI were also related to poor outcomes, whereas higher DLCO and higher BMI were related to favorable outcomes among SSc (Table 3).

## Discussion

Although microstomia is one of the most characteristic features regarding systemic sclerosis, there is no standardized methodology on how to assess oral aperture and which parameters are beneficial during the patients’ follow-up.

In published literature, the measurement of interincisal distance was most frequently used to examine the MOA among SSc patients [17–21]. Reduced ID was associated with overall disease severity, the presence of anti-topoisomerase I autoantibodies, internal organ involvement, more extended skin involvement and higher malnutrition risk [17, 42, 43]. One recent French study has also shown dcSSc patients with a high ID at baseline yet which shows a decrease during follow-up were at risk of poor survival and development of ILD [24]. LD was found to correlate with HAQ-DI in SSc [26]. In another study, reduced LD was weakly associated with lower FVC [44].

There are data available regarding MOA and its influencing factors, including age, race, and sex in different countries [45, 46]. For a reliable comparison in reference to SSc patients, we assessed the MOA among healthy Hungarian individuals in our study based on the same protocol we used among SSc patients. We examined both the vertical and horizontal width of oral aperture and we also created two new composite measures, the oral area and oral circumference.

Although all MOA parameters among the female HCs were significantly lower than the values of male HCs, we found no statistical difference in ID and LD between the two sexes among SSc patients. The lack of difference in vertical parameters between sexes is compatible with the same observation carried out by Baron et al., in which the study population was similar to our cohort [42]. Our results suggest male patients are more severely affected by the decrease of MOA than when compared to females.

Microstomia occurred in 74 patients (56%) in our study, which was slightly less frequent than the prevalence found in a review study in a total of 368 SSc patients (69.8%), which used a less strict cut off value of ID < 40 mm [47]. As expected, the mean values of the vertical parameters (ID, LD) as well as the two novel OA and OC values were lower in the entire SSc cohort when compared to the controls. The decrease of the ID, LD, OA, and OC occurred within the first two years following disease onset, showing the decrease of MOA can be an early manifestation in a subset of patients. Although decreased ID was more prevalent among dcSSc patients, decreased LD and OA was similarly frequent in both the diffuse and limited SSc, demonstrating in our patient cohort, lcSSc patients were also significantly affected by microstomia on the contrary with previous data in published literature [48].

Regarding the association of MOA parameters and clinical variables, in our study, ID among dcSSc patients with disease duration > 2 years showed moderate correlation with BMI and negative correlation with mRSS, whereas LD and OA correlated moderately negatively with ESR in this patient subset, potentially reflecting an ongoing inflammation-related disease activity among patients with decreased MOA parameters.

Additionally, we found strong associations between variables reflecting structural damage of the joints (presence of contractures and decreased hand anatomic index) and different MOA parameters among patients with more than 2 years of disease duration. Decrease in ID was associated with CCs only among dcSSc patients, whereas diminished LD, OA and OC were related to anatomical damage in both the limited and diffuse subsets. Therefore, the measurement of LD has an added value when compared to ID in the identification of those lcSSc patients, who are at higher risk to develop structural involvement of the joints.

Furthermore, the ID, LD, OA, and OC values were also associated with the HAQ-DI reflecting global functional disability. Decreased MOA values already manifested within 2 years of disease onset in a subset of patients. HAQ-DI was correlated with MOA values during both the early phase and at longer disease duration.

Although the preliminary EScSG-AI available at the time of the enrollment did not show any relationship with the MOA parameters, patients with active disease based on the Pecs-AI (Pecs-AI ≥ 2.5) had lower LD values upon enrollment. Decreased LD was more frequently associated with an active disease in the entire SSc cohort and among lcSSc patients.

These results reflect the premise in which decreased mouth opening identifies a subset of patients who are at higher risk for overall functional disability during early disease course and the appearance of structural damage in the joints following > 2 years of disease duration. The overall disability, decrease in MOA and development of joint contractures also parallel with persistent disease activity and inflammation.

Only a few data are available regarding the natural course of MOA and the clinical significance of microstomia in the long-term follow-up. MOA was found to be mostly stable over time in two recent studies. Additionally, microstomia defined as ID < 30 mm was present in 7–17% of patients at the time of enrollment [17, 24]. In the Dutch cohort, the association between decreased ID over time (follow up time of 2 years) and more severe organ involvement was described [17]. The French national cohort study, using trajectory models among 349 SSc patients demonstrated 9.5% of the patients had high yet decreasing ID (cluster 1), 88.8% had baseline ID > 35 mm which stabilized over time (cluster 2), and 1.7% had a low baseline ID increasing over time (cluster 3) [24]. Patients in cluster 1, belonging more frequently to the dcSSc subset, were at risk of poor survival and development of ILD [24].

Although this latter study described for the first time the relationship between poor survival and decrease in ID among dcSSc patients based on univariate analysis, to our knowledge, our study is the first report on the effect of microstomia regarding long term survival among SSc based on multivariate regression analysis, assessing all potential MOA parameters and previously identified poor prognostic variables.

Based on the Kaplan-Meier survival analysis, in addition to the well-known poor prognostic factors, decreased LD, OA and OC values, yet not ID upon enrollment, were also associated with diminished survival outcomes. Based on the multivariate regression analysis only male sex, decreased oral area, higher HAQ-DI, ESR > 30 mm/h and higher age upon disease onset were independent poor prognostic factors regarding long-term survival, whereas higher DLCO values and higher BMI were associated with a more favourable outcome. Decreased OA carried a 2.74 times higher risk for all-cause mortality in the entire SSc cohort independently from the other formerly described classical poor prognostic factors. Decreased OA identified both lcSSc and dcSSc patients with advanced microstomia and worse overall survival rates, therefore, we recommend the use of this variable in the evaluation of prognosis in the entire SSc patient cohort.

Several other previously identified independent poor prognostic factors (e.g., dcSSc subset, cardiac involvement, joint contractures, low FVC and scleroderma renal crisis) were not identified as poor prognostic factors in our study. This can be explained by the fact in which some organ involvements were infrequent within our patient cohort (ejection fraction < 50%, kidney involvement). Additionally, patients with a relatively lengthy disease duration at the time of enrollment (median 6 years) coupled with a lengthy disease follow-up period (median 12.5 years) may favor the bias in which milder cases are over-represented. Furthermore, we did assess only the organ manifestations at the time of enrollment, not the newly developed ones during follow-up, therefore, the prevalence of different internal organ involvements at the time of the demise is under-estimated. However, the identification of well-known poor prognostic factors in addition to OA and its relative high risk (HR: 2.74, 95% CI: 1.15–6.53) in our multivariate analysis model makes this model plausible.

We are aware of further limitations of our study. Although we have created a HC group with similar median age and sex distribution, the number of participants was relatively low, therefore, their results cannot be generalized to the entire Hungarian population. The enrollment of a higher number of volunteers and determination of population based upon normal ranges is needed in further multicenter studies.

Distinctively, the strength of our study is the inclusion of healthy controls, therefore, the cut-off values based on these results reflect more precisely the normal range values of MOA among SSc patients when compared to a previously arbitrarily defined strict value. Although the median age of SSc patient group was higher than in the HCs, there was no statistically significant difference in age-, and sex-distribution between the patient and control group therefore we assume that this did not significantly influence the calculation of eligible cut-off values.

In conclusion, our findings demonstrate that a large proportion (56%) of SSc patients are affected by reduced mouth opening involving both the dcSSc and lcSSc subsets. Microstomia can be present in early disease phase (≤ 2 years disease duration). Patients with decreased MOA suffer more damage in the small and large joints, have higher overall disability and experience more frequently active disease, potentially due to an ongoing inflammation. The beneficial, convenient measurable interlabial distance has an added value to interincisal distance in identifying both dcSSc and also lcSSc patients with higher structural joint damage burden and increased overall disability. Therefore, LD is a useful parameter to be assessed during the follow-up. The oral area is an independent poor prognostic factor regarding worse long-term survival rates among SSc patients. Based on our results, the two most valuable parameters of oral aperture in SSc are the interlabial distance and oral area, which are associated with increased disability (LD) and poor prognosis (OA).

## Electronic supplementary material

Below is the link to the electronic supplementary material.

Supplementary Material 1

## Data Availability

The datasets generated and/or analysed during this study are not publicly available due to ethical issues but are available from the corresponding author on reasonable request.

## Abbreviations

BMI: Body Mass Index
CC: Joint contracture
CI: Confidence interval
COVID-19: Coronavirus 2019 pandemic
CW: Intercommissural width at closed mouth
dcSSc: Diffuse cutaneous systemic sclerosis
DLCO: Diffusing Capacity Of The Lungs For Carbon Monoxide
EScSG-AI: European Scleroderma Study Group Activity Index
ESR: Erythrocyte sedimentation rate
extCC: Extensive contracture
FVC: Forced vital capacity
GP: General practitioner
HAI: Hand Anatomic Index
HAQ-DI: Health Assessment Questionnaire Disability Index
HC: Healthy control
HR: Hazard Ratio
ID: Interincisal distance
IQR: Interquartile range
lcSSc: Limited cutaneous systemic sclerosis
LD: Interlabial distance
LLN: Lower limit of normal
MOA: Mouth opening ability
OA: Oral area
OC: Oral circumference
OW: Intercommissural width at opened mouth
Pecs-AI: Scleroderma Activity Index derived from the EScSG-AI
QoL: Quality of Life
ROM: Range of motion
SD: Standard deviation
SSc: Systemic sclerosis
