# Supplementary material for: Microstomia is associated with functional impairment and is a poor prognostic factor in systemic sclerosis – a single center observational study with survival analysis
Source: BMC Oral Health. 2024 Nov 15;24:1390. doi: 10.1186/s12903-024-05178-6 (PMC11568575; doi:10.1186/s12903-024-05178-6)
Supplement: Supplementary file 1 — Supplementary Material 1 [file 12903_2024_5178_MOESM1_ESM.docx]

**Additional File Figure 1.** Measured and derived oral aperture parameters based on the formulas of the ellipse

**
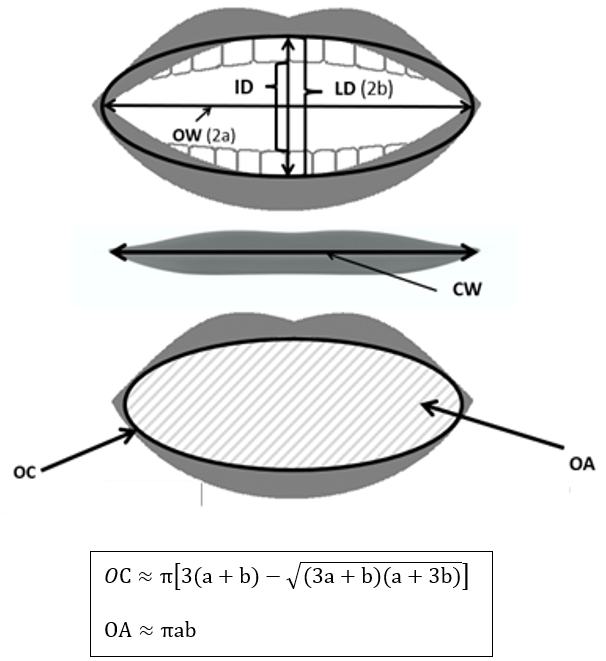
**

*Legend: ID: vertical interincisal distance at maximally opened mouth; LD: vertical interlabial distance at maximally opened mouth; OW: horizontal width at maximally opened mouth; CW: horizontal width at closed mouth; OA: oral area; OC: oral circumference; ‘a’ is the semi-major axis of the ellipse calculated as half of the measured OW; ‘b’ is the semi-minor axis of the ellipse calculated as half of LD.*

**Additional File Table 1.** Comparison of oral aperture parameters between the healthy control group and the systemic sclerosis patients grouped by sex

|  | Healthy control group | | Systemic sclerosis patients | |
| --- | --- | --- | --- | --- |
|  | Female (n=55) | Male (n=8) | Female (n=119) | Male (n=12) |
|  | Mean ± SD | Mean ± SD | Mean ± SD | Mean ± SD |
| ID (mm) | 46 ± 7 **^a, c^** | 51 ± 4 **^c^** | 32 ± 7 | 37 ± 10 |
| LD (mm) | 50 ± 6 **^a, c^** | 60 ± 4 **^c^** | 39 ± 8 | 44 ± 10 |
| CW (mm) | 50 ± 5 **^a^** | 59 ± 6 | 50 ± 6 **^b^** | 56 ± 12 |
| OW (mm) | 45 ± 5 **^a, c^** | 53 ± 4 **^c^** | 43 ± 6 | 48 ± 6 |
| OA (mm2) | 1819 ± 443 **^a, c^** | 2475 ± 316 **^c^** | 1343 ± 370 **^b^** | 1729 ± 586 |
| OC (mm) | 151 ± 18 **^a, c^** | 176 ± 11 **^c^** | 130 ± 17 **^b^** | 146 ± 24 |

*Legend:*

*ID: vertical interincisal distance at maximally opened mouth; LD: vertical interlabial distance at maximally opened mouth; OW: horizontal width at maximally opened mouth; CW: horizontal width at closed mouth; OA: oral area; OC: oral circumference;*

***^a^-*** *comparison based on sex in the healthy control group (Mann-Whitney U test, p<0.05)*

***^b^****- comparison based on sex in the systemic sclerosis patients (Mann-Whitney U test, p<0.05)*

***^c^****- comparison of the corresponding sexes between the healthy control group and the systemic sclerosis patients (Mann-Whitney U test, p<0.05)*
